# Supplementary material for: The interactome of intact mitochondria by cross-linking mass spectrometry provides evidence for coexisting respiratory supercomplexes
Source: Mol Cell Proteomics. 2017 Dec 8;17(2):216–32. doi: 10.1074/mcp.RA117.000470 (PMC5795388; doi:10.1074/mcp.RA117.000470)
Supplement: Supplemental Data [file supp_RA117.000470_133922_0_supp_23978_fzffwf.zip › spectra_annotation/mito_spectra_annotation/221-1-16-1-21-1.pdf]

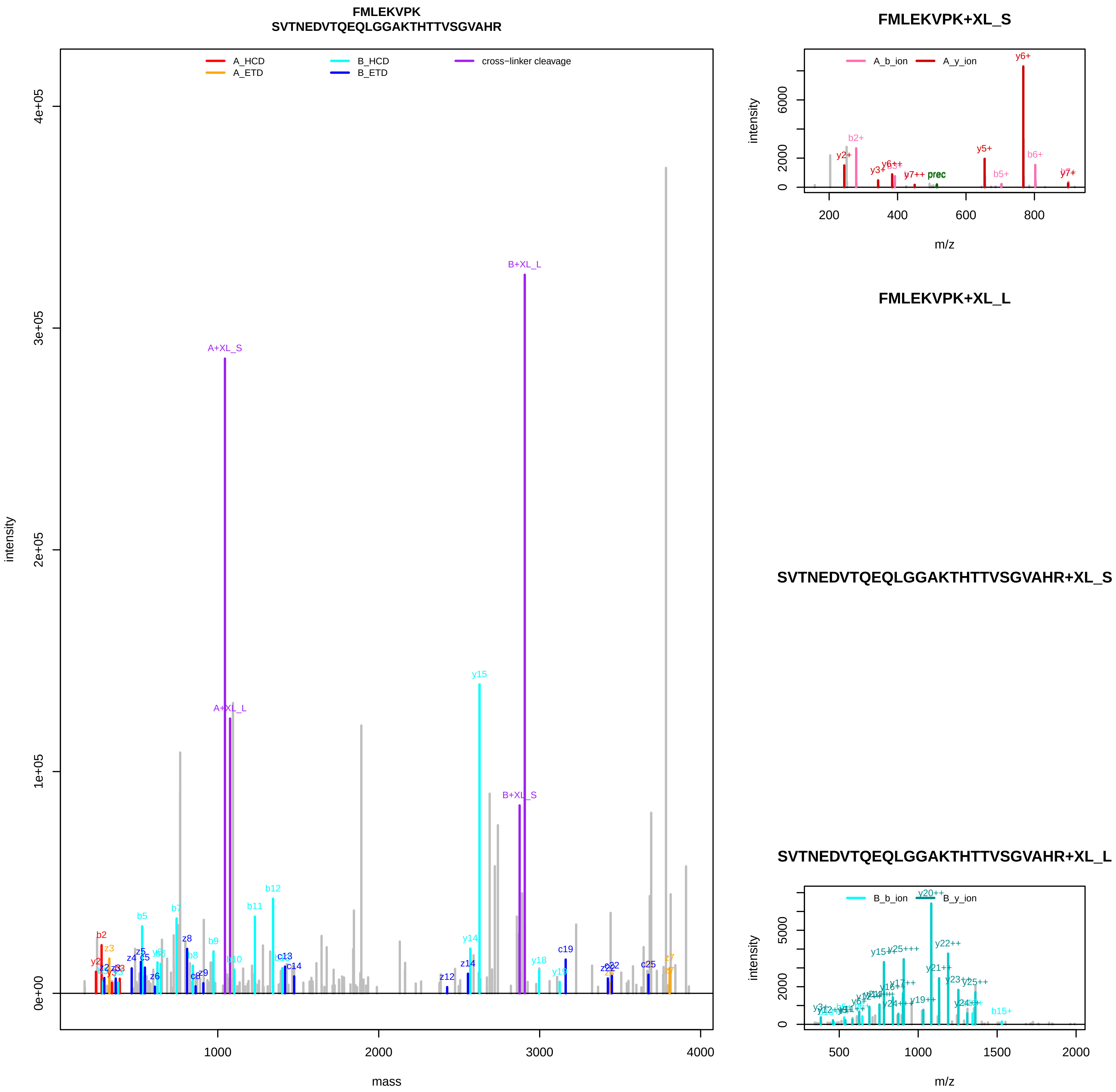
FMLEKVPK
SVTNEDVTQEQLGGAKTHTTVSGVAHR
A_HCD
A_ETD
B_HCD
B_ETD
cross−linker cleavage
A+XL_S
A+XL_L
B+XL_L
B+XL_S
intensity
mass
0e+00
1e+05
2e+05
3e+05
4e+05
1000
2000
3000
4000
FMLEKVPK+XL_S
A_b_ion
A_y_ion
m/z
FMLEKVPK+XL_L
SVTNEDVTQEQLGGAKTHTTVSGVAHR+XL_S
SVTNEDVTQEQLGGAKTHTTVSGVAHR+XL_L
B_b_ion
B_y_ion
